# Corns Cured

**Published:** 1859-12

**Authors:** 


### CORNS CURED.

The safest, the most accessible, and the most efficient cure of a corn on the toe, is to double a piece of thick soft buckskin, cut a hole in it large enough to receive the corn, and bind it around the toe. If, in addition to this, the foot is soaked in warm water for five or more minutes every morning and night, and a few drops of sweet or other oily substance are patiently rubbed in on the end after the soaking, the corn will almost infallibly become loose enough in a few days to be easily picked out with the finger nail; this saves the necessity of paring the corn, which operation has sometimes been followed with painful and dangerous symptoms. If the corn becomes inconvenient again, repeat the process at once.

